# Kin recognition for incest avoidance in Damaraland mole-rats, *Fukomys damarensis*

**DOI:** 10.1098/rspb.2024.1138

**Published:** 2024-10-16

**Authors:** Amy E. Leedale, Philippe Vullioud, David Seager, Markus Zöttl, Gaétan Glauser, Tim Clutton-Brock

**Affiliations:** ^1^ Department of Zoology, University of Cambridge, Cambridge, UK; ^2^ Kalahari Research Centre, Kuruman River Reserve, Northern Cape, South Africa; ^3^ School of Science, Engineering & Environment, University of Salford, Salford, UK; ^4^ Neuchâtel Platform of Analytical Chemistry, University of Neuchâtel, Neuchâtel, Switzerland; ^5^ Department of Biology and Environmental Science, Linnaeus University, Växjö, Sweden; ^6^ Mammal Research Institute, Department of Zoology and Entomology, University of Pretoria, Pretoria, South Africa

**Keywords:** kin recognition, kin discrimination, inbreeding, cooperative breeder, reproductive activation, ovulation

## Abstract

Across taxa, breeding among close relatives is usually avoided because it incurs fitness costs to offspring. Incest is often averted through the dispersal of either sex from the natal area to breed. In some philopatric species, association among relatives extends into adulthood, and an ability to discriminate kin may be required for individuals to reduce inbreeding risk. Here, we aim to determine the mechanism of kin recognition for incest avoidance in the Damaraland mole-rat *Fukomys damarensis*, a cooperative breeder characterized by extreme reproductive skew. Pairs of opposite-sex adults were formed in the laboratory and, within pairs, genetic relatedness and degree of familiarity were manipulated through cross-fostering experiments. We found that unfamiliar pairs were more likely to engage in sexual behaviours and bred more successfully than familiar pairs, regardless of their genetic similarity. Females paired with unfamiliar males were also more likely to exhibit reproductive activation, characterized by increased levels of oestradiol and progesterone. This study shows that in Damaraland mole-rats, inbreeding avoidance can be achieved through a discrimination mechanism that relies on association during rearing, and that ovulation is induced by mating. This study advances our understanding of incest avoidance in species with constrained dispersal.

## 1. Background

The negative effects of inbreeding on offspring fitness have been documented across a wide range of taxa (e.g. [1–3]). Such costs, referred to as inbreeding depression, have led to the evolution of various mechanisms of inbreeding avoidance among a diversity of organisms including plants [4] arthropods [5] and vertebrates [6]. Inbreeding depression typically arises through the unmasking of harmful recessive alleles which, when expressed, result in traits that reduce fitness [7]. In habitually inbred populations, harmful recessives can be purged from the genome through selection [2,8], and the extent of inbreeding depression between populations can vary. When inbreeding costs are outweighed by the costs of delayed or missed opportunities for reproduction, inbreeding may be tolerated [9]. Inbreeding can also be adaptive, through increased relatedness to offspring, and increased reproductive success of relatives [10]. Thus, whether incest is avoided, tolerated or preferred depends on the balanced fitness consequences of inbreeding, and the risk of mating with a relative [11,12]. In nomadic species, the risk of inbreeding can be alleviated through natal dispersal of one or both sexes, which effectively separates relatives in space or time [13,14]. In contrast, delays or constraints to dispersal, which create an extended period of association among related adults, selects for alternative means to avoid costly inbreeding in sedentary or philopatric species [15,16].

Despite the potential risk of inbreeding in such species, mating among relatives is typically avoided, either by recognition and subsequent rejection of kin during mate choice [17–19] or by post-copulatory rejection of a relative’s gametes [20]. Kin recognition may be based on familiarity, whereby individuals learn the phenotypes of those they associate with during early life, and subsequently treat these familiar individuals as kin [21]. Alternatively, conspecifics whose phenotypic similarity exceeds a certain threshold are considered kin, regardless of any prior association [22]. Familiarity is an effective mechanism when individuals encountered in a certain context are likely to be kin, such as brood mates sharing a nest, whereas phenotype matching relies on a stable correlation between phenotypic similarity and genetic relatedness. In practice, teasing apart these mechanisms has proved difficult; their use may be context-dependent [23], and there is some evidence to suggest that both mechanisms can operate within species [24]. Investigations into the ontogeny of kin recognition require carefully designed experiments that dissociate genetic relatedness and familiarity in functionally relevant contexts.

Using captive Damaraland mole-rats *Fukomys damarensis*, a cooperatively breeding African mole-rat (family: Bathyergidae), we experimentally investigate the mechanism of kin recognition that permits avoidance of relatives as mates, and the fitness consequences of incestuous pairing in this species. Damaraland mole-rats live in large groups, characterized by an extreme reproductive skew and low rates of dispersal [25,26]. Groups typically comprise a single breeding pair and their non-breeding offspring from several generations [6,27]. Unlike another highly social Bathyergid, the naked mole-rat, *Heterocephalus glaber*, territory inheritance is rare and groups usually fragment or quiesce after the death of one or more breeders [27]. Immigration into breeding groups is rare, and there is good evidence that subordinate female reproduction is limited by access to unrelated males: subordinate females that lack access to unrelated males abstain from breeding, even when the dominant female is absent, but will attempt to mate with introduced males [28–30]. Indeed, females appear only to ovulate or ‘activate’ their reproductive axis upon encountering an unrelated male [29,31]. Although the role the dominant female plays in suppressing subordinate reproduction cannot be excluded [32,33], these findings suggest that incest avoidance maintains extreme reproductive skew in Damaraland mole-rats. Such strong inbreeding avoidance, despite the reproductive cost of lost breeding opportunities among subordinates, suggests that inbreeding carries substantial fitness costs. One question that remains is how relatives are recognized. Together, these life-history traits make Damaraland mole-rats an ideal subject to investigate kin recognition mechanisms in the context of mating decisions.

Kin recognition may operate through prior association in early life or an assessment of relatedness through phenotype matching. Damaraland mole-rats behave aggressively to colony mates after a period of separation [34], and several studies have shown that familiar, close relatives are avoided as mates [28,35]. But, few studies effectively separate the role of familiarity and genetic relatedness for kin recognition in the context of mating decisions. Two recent studies have begun to tease apart these mechanisms. Carter *et al.* [36] found that siblings separated for five weeks before pairing readily mated. Later, Kelley *et al.* [37] showed that when pairs of unfamiliar non-relatives were introduced, but restricted from physical contact for two weeks, they abstained from mating thereafter. This suggests that frequent association, and the context of initial encounters, is important for kin recognition. However, the ontogeny of kin discrimination, as well as the relative importance of kinship and early environment, remain inconclusive.

Here, we use cross-fostering experiments to determine how mating behaviour, reproductive physiology and reproductive success are affected by (i) association during rearing (familiarity) and (ii) genetic relatedness (kinship). We aim to investigate: (i) the ontogeny of kin discrimination, by determining the relative roles of kinship and familiarity on mating decisions; (ii) the effect of kinship and familiarity on female reproductive physiology; and (iii) how these variables influence reproductive success. These are tested by measuring the response of females to assigned males, which vary in both kinship and familiarity. We hypothesize that incest avoidance relies predominantly on familiarity during rearing and predict that mating behaviour will be observed more frequently within unfamiliar pairs than familiar pairs, regardless of kinship. We also predict that females paired with unfamiliar males will exhibit reproductive activation, whereas those paired with familiar males will exhibit no activation. Finally, we predict lower reproductive success among incestuous pairs than unrelated pairs.

## 2. Methods

### (a) Study animals and husbandry

Data were collected from a captive population of Damaraland mole-rats in the Kuruman River Reserve, Northern Cape, South Africa. The captive population originated from a wild population of 242 individuals trapped in the reserve and surrounding area in 2013 (mean ± s.d. group size = 8.16 ± 5, range = 2–26). Since 2013, the pairing of unrelated individuals has expanded the captive population to 554 mole-rats (mean ± s.d. group size = 5.5 ± 4.4, range = 1–17). In this experiment, most subjects (77%) were sired by wild caught individuals (first generation), whereas 17% had one lab-born parent (second generation) and 6% had two lab-born parents (second generation). Groups were housed in standardized, self-contained tunnel systems of polyvinyl chloride (PVC) pipe, with windows of transparent plastic. Each tunnel system comprised vertical pipes through which fresh sand was provided daily, a sand waste box, nest box, toilet and a food store. Animals were provisioned ad libitum on a diet of sweet potatoes and cucumbers, twice daily. Individuals were identified using a unique coloured dye mark applied to their head patch and a passive integrated transponder (PIT) tag implanted in early life.

### (b) Experimental design

Pairs were formed by placing two non-breeding, sexually mature individuals of the opposite sex into new, self-contained tunnel systems. Mole-rats were considered sexually mature at 15 months, and weighing at least 80 g for females and 100 g for males. Opposite-sex pairs were approximately matched by age and weight. Before pairing, subjects were removed from their colonies and placed in isolation with food, sand and enrichment (a section of PVC pipe and shredded tissue paper) for 24 h to simulate emigration.

The experiment was initially carried out on 32 pairs of opposite-sex individuals that were either (i) familiar kin (*n* = 8); (ii) familiar non-kin (*n* = 8); (iii) unfamiliar kin (*n* = 8); or (iv) unfamiliar non-kin (*n* = 8), in a full factorial treatment design. One pair of unfamiliar kin was compromised by an extra-group copulation following an escape, resulting in pregnancy, leaving seven pairs in the unfamiliar kin treatment (*n* = 31 pairs). Pairs of familiar kin comprised opposite-sex nest mates, reared in their mutual, natal colony until pairing. Pairs of unfamiliar kin were formed of opposite-sex nest mates, transferred to separate foster colonies 9.71 ± 4.21 (mean ± s.d.) days after birth, in which they were reared until pairing. Pairs of familiar non-kin were formed of unrelated individuals born in separate natal colonies, transferred to the same foster colony 8.13 ± 1.82 days after birth, in which they were reared until pairing. Pairs of unfamiliar non-kin were formed of unrelated individuals, reared separately in their respective natal colonies until pairing. Cross-fostered pups were transferred in groups of 2–5 pups, and readily accepted by 97% of foster colonies. Treatments (ii) and (iii) included foster subjects (*n* = 32) from 18 foster colonies. The mean genetic relatedness within pairs is summarized by treatment in table 1.

**Table 1.** Mean ± s.d. genetic relatedness of opposite-sex pairs [38].

treatment	coefficient of relatedness (*r*)
familiar kin (*n* = 8)	0.440 ± 0.174
unfamiliar kin (*n* = 7)	0.435 ± 0.094
familiar non-kin (*n* = 8)	0.016 ± 0.192
unfamiliar non-kin (*n* = 8)	−0.033 ± 0.118

### (c) Genetic analyses

The genetic relatedness between pairs was estimated using Queller & Goodnight’s [38] coefficient of relatedness, *r*, in SPAGeDi v. 1.1.5 [39]. This relatedness estimate has been found to be reliable when tested against known relationships (mother-offspring). DNA was extracted from tissue and amplified. Individuals were genotyped at 13 autosomal microsatellite loci: DMR2-5, 7, CH1-3, LV25, NCAM [32,40], Cmech3, 4 and 6 [41]. Population allele frequencies were generated using all genotyped individuals (*n* = 474) in CERVUS v. 3.0.7 [42], to maximize accuracy in estimating rare allele frequency and ensure non-zero allele frequencies.

### (d) Behavioural observations

Behavioural observations were carried out to quantify mating behaviour, focusing on copulation, defined as one individual mounting another and attempting intromission with pelvic thrusts, and sex foreplay, defined as the rapid succession of bites, sparring, sniffs, passes and drumming. A full ethogram of these and other observed mole-rat behaviours are presented in electronic supplementary material, table S1A. Behavioural observations consisted of focal and scan sampling. Focals were carried out on the female. One 2 h focal session was carried out immediately after pairing (day 0, approx. 10.00 SAST), and another 1 h focal session the following day (day 1, approx. 08.00 SAST). Focal behaviours were sampled as ‘states’, recorded with a start and an end time, or ‘events’, recorded at observation without a duration (electronic supplementary material, table S1B). Weekly 12 h scan sessions were carried out for eight weeks, starting 2–8 days after pairing (approx. 07.00 SAST). Four pairs were observed concurrently during each session (*n* = 8 individuals). Scan sessions comprised a combination of instantaneous and continuous sampling (electronic supplementary material, table S1C). Behavioural states were recorded every 4 min, generating 180 instantaneous samples per individual. In between instantaneous sampling, events and states of short duration were recorded continuously. For both focals and scans, observations were recorded using Observer 11XT pocket v. 3.2.

### (e) Reproductive physiology

Urine samples were collected to quantify oestradiol (E2) and progesterone (P4) and determine the effect of treatment on reproductive activation. Samples were collected 2–4 days before pairing to establish baseline E2 and P4 levels. Samples were subsequently collected on day 1, then every 3 days until day 90 and every 7 days between day 90 and day 270. E2 and P4 levels were quantified using high-performance liquid chromatography-tandem mass-spectrometry (electronic supplementary material, S2).

For efficiency, a subset of samples were carefully selected for hormone analyses. Overall, we used gestation, abortion, parturition and endocrine data to select samples that provided the greatest resolution in the timing of reproductive activation, detected by ovulation-induced increases in E2 and P4. For all females, samples collected between 2 days before pairing and 60 days after pairing were initially selected to determine whether reproductive activation occurred. Reproducing females were sampled every 3 days, which included samples collected immediately after activation and during the first trimester of successful gestation, or over a similar time period of luteal phases or aborted gestation, to assess the duration of induced elevations in E2 and P4 post-ovulation. Females that did not reproduce were sampled every two weeks: this duration was shorter than observed post-ovulation increases E2 and P4, ensuring reproductive activation could not be missed. If reproductive activation was detected in non-reproducing females, sampling frequency was increased to match that of reproducing females. To verify that females that did not activate their reproductive axis within 60 days of pairing had still not ovulated by the end of the experiment, we selected additional samples between 210 and 270 days post-pairing, at a frequency of 7–14 days.

To support the interpretation of hormonal profiles, we used gestation length estimates calculated from previous breeding events for which the exact conception time is known. Mean estimated gestation length is 96.3 ± 3.2 days (P Vullioud, 2024, unpublished data, *n* = 3). Note that this represents a maximum duration, as fertilization may occur a few days after mating. As it was not possible to determine first ovulation using pre-ovulatory surges in E2, reproductive activation was determined based on threshold levels of E2 and P4. To minimize subjectivity, we developed a series of threshold-based criteria (*n* = 10), which were used to separately assess reproductive activation (electronic supplementary material, S2).

### (f) Reproductive success

All pairs were closely monitored for nine months following pairing. To investigate reproductive success, we tested the effect of treatment on the (i) probability of successful gestation following reproductive activation; (ii) total number of pups produced; and (iii) total number of litters produced.

### (g) Statistical analysis

All data analyses were performed in R v. 4.2.1 [43], using Generalized Linear Mixed Models (GLMMs) specified in the *glmmTMB* package [44]. To determine the significance of pairwise differences between treatments post hoc, we performed analyses of deviance (Wald *χ*^2^ test) with Tukey HSD adjustment on estimated marginal means with the *emmeans* package [45]. Observations of sexual behaviour were compared across treatments using tweedie GLMMs with rates of copulation and sex foreplay specified as response variables. Rates were analysed as counts/hour for focal data and counts/12 h for scan data, to account for variation in the duration of observation sessions (electronic supplementary material, S1D–E). Scan counts were computed over the total duration during which behaviours could be recorded (total session duration minus time taken to record instantaneous sampling, mean ± s.d. continuous sampling duration = 198.22 ± 44.26 min, range = 116.15–309.8). Pair and session ID were included as random effects, to account for multiple observations of pairs. We compared hormone levels within 60 days of pairing across treatments using tweedie GLMMs, with E2 and P4 specified as response variables and female ID as a random effect. For P4, we specified the model to estimate a dispersion parameter for each treatment to avoid issues of heteroscedasticity in the residuals.

To investigate reproductive success, we compared the timing of reproductive activation between treatments. Two females from the unfamiliar non-kin treatment took over twice as long to activate their reproductive axis than the next longest female in this treatment (electronic supplementary material, figure S4E), so to consider the possibility that these may be outliers, models were sequentially ran with none, one and both of these potential outliers removed. We used generalized poisson (all data) and gamma (outliers removed) GLMMs with log link and specified the number of days between pairing and reproductive activation as the response variable (one model for each activation criterion). We also compared the likelihood of successful gestation between treatments, specified in a binomial GLMM as whether females produced their first litter within one hundred days of activation. Finally, the number of pups and litters produced was compared using poisson GLMMs with Pair ID specified as a random effect.

## 3. Results

### (a) Behavioural observations

**Figure 1.**
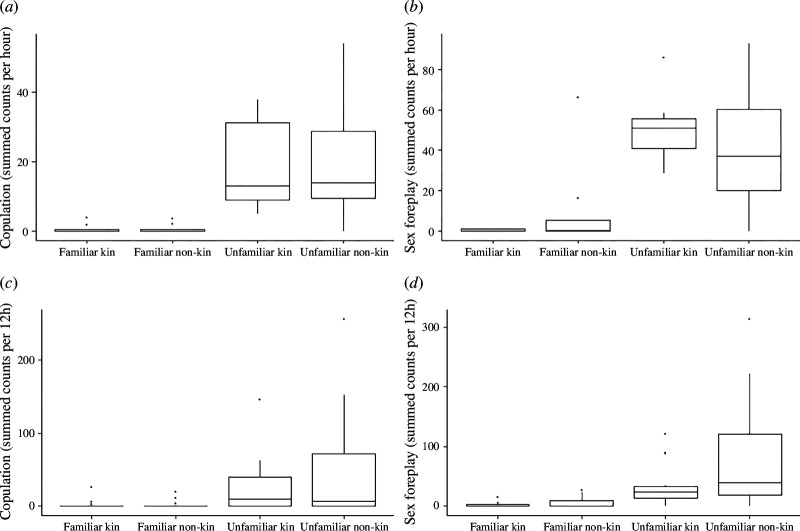
Total counts of sexual behaviour observed during focal observations (*a–b*) and scan observations (*c–d*) of opposite-sex pairs of Damaraland mole-rats that are familiar kin (*n* = 8), familiar non-kin (*n* = 8), unfamiliar kin (*n* = 7) or unfamiliar non-kin (*n* = 8). Focal observations were carried out on the female for approximately 3 h across two sessions. Scan sessions were carried out weekly for eight weeks. During scans, individuals of both sex were observed for approximately 12 h per session. Counts from both focals and all scans are summed. Boxes and whiskers represent within-treatment variation among pairs. Boxes represent the inter-quartile range (IQR). Whiskers extend to ±1.5 IQR. Line across the box indicates the median. Outliers are represented by individual data points.

Analyses of focal data revealed a significant effect of treatment on the rate of copulation (*χ*^2^ = 20.35, d.f. = 3, *p* < 0.001; figure 1*a*) and sex foreplay (*χ*^2^ = 30.43, d.f. = 3, *p* < 0.001; figure 1*b*; electronic supplementary material, table S3A). Post hoc analyses revealed increased rates of both behaviours among unfamiliar pairs, compared with familiar pairs, whereas kinship had no effect on either copulation or sex foreplay (electronic supplementary material, table S3B). The scan observations showed similar results to the focal observations, with a significant effect of treatment on the rate of copulation (*χ*^2^ = 35.57, d.f. = 3, *p* < 0.001; figure 1*c*) and sex foreplay (*χ*^2^ = 37.03, d.f. = 3, *p* < 0.001; figure 1*d*; electronic supplementary material, table S3C). As with the focal data, post hoc analyses revealed greater copulation and sex foreplay rates among unfamiliar pairs, compared with familiar pairs, with no effect of kinship on either behaviour (electronic supplementary material, table S3D). In the focal and scan observations, counts of sexual behaviour among familiar pairs were close to zero (figure 1).

### (b) Reproductive physiology

**Figure 2.**
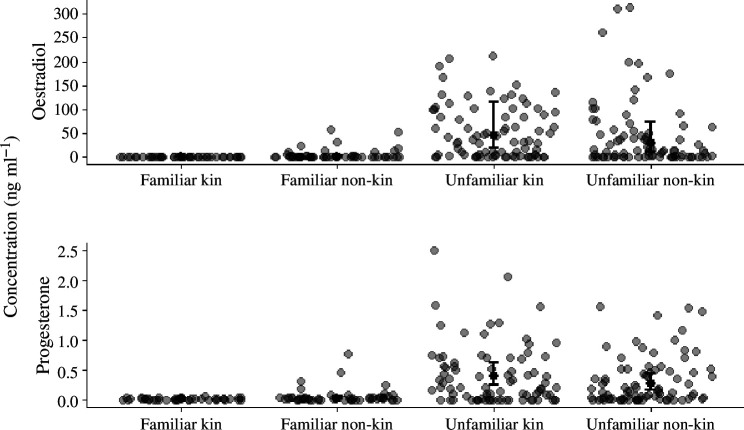
Differences in oestradiol (upper panel) and progesterone levels (lower panel) between females paired with a familiar (left panel, *n* = 16) and an unfamiliar male (right panel, *n* = 15). Solid dots indicate predicted treatment level concentrations at the response scale obtained by back transforming the fixed effects of tweedie GLMMs with log-link. Solid lines indicate 95% CIs (fixed effect ±1.96 s.e.). Grey dots indicate hormone concentrations of urine samples that were used for statistical analyses. To facilitate visualization, one data point with a concentration of >500 ng ml^−1^ of E2 has been removed.

Visual inspection of E2 and P4 profiles showed that both hormones remained low in 94% (15/16) of females paired with familiar males (electronic supplementary material, figure S2D). In contrast, E2 and P4 started rising within a few days or weeks of pairing in 93% (14/15) of females paired with unfamiliar males and remained elevated for several weeks, a hormone profile associated with early gestation in eight individuals (electronic supplementary material, figures S2D and S4A). Both E2 and P4 were significantly higher in females paired with unfamiliar males than in females paired with familiar males (electronic supplementary material, tables S4C–D). Among non-kin, E2 was 4.09 times higher in females from unfamiliar pairs compared with those from familiar pairs. Among kin, E2 was 126.77 times higher in females from unfamiliar pairs compared with those from familiar pairs. Similar elevations were observed in P4, which was 4.74 times higher in females paired with unfamiliar non-kin compared with familiar non-kin, and 19.29 times higher in females paired with unfamiliar kin, compared with familiar kin (figure 2). As with the behavioural observations, kinship had little effect on hormone levels (electronic supplementary material, tables S4C–D).

Overall, there was no conclusive effect of kinship on the timing of reproductive activation of females that were paired with an unfamiliar male (electronic supplementary material, figures S4E–H). The difference in the timing of reproductive activation only reached significance for one out of ten reproductive activation criteria after exclusion of the two outliers (1/30 models; electronic supplementary material, figure S4H). Under this criterion, females paired with an unrelated male activated their reproductive axis 6.38 days earlier than females paired with a relative.

### (c) Reproductive success

None of the females paired with familiar males produced litters, whereas all females paired with unfamiliar males produced at least one litter. Within females that produced litters, kinship did not affect the number of pups (estimate = 0.02 ± 0.33, *z* = 0.08, *p* = 0.94) or litters produced (estimate = −0.02 ± 0.34, *z* = −0.05, *p* = 0.96; electronic supplementary material, table S5A), or the likelihood of successful gestation following activation (estimate = 1.20 ± 1.13, *z* = 1.06, *p* = 0.29, electronic supplementary material, table S5B).

## 4. Discussion

Our experiment demonstrates that without prolonged association after birth, Damaraland mole-rats will readily mate with a relative. Indeed, when provided the opportunity to do so, opposite-sex pairs of unfamiliar kin breed at a similar frequency to unfamiliar non-kin. This suggests that familiarity comprises an important component of kin recognition for inbreeding avoidance in this species. In contrast, we observed strong incest avoidance within opposite-sex pairs reared together, with consistently low rates of sexual activity, regardless of kinship. Our findings suggest that in the context of mating decisions, the ontogeny of kin recognition in Damaraland mole-rats depends on association during early life; augmenting studies that manipulate familiarity among mature relatives to show that association must be maintained [36,37]. As our experimental subjects were separated at approximately 10 days after birth, any learning of kin during this window seems not to be implemented in the decision to mate with opposite-sex conspecifics later in life. Kin recognition mechanisms mediated by familiarity appear to operate in other species in which there is a risk of inbreeding and individuals encountered during certain life stages are likely to be kin [15,46]. This contrasts with some other social species, in which phenotype matching without prior association appears to be the most likely mechanism of kin recognition [47,48]. For example, phenotype matching for incest avoidance has been demonstrated in communally breeding house mice *Mus musculus* [49]. In such systems, association during early rearing conditions may not be a reliable indicator of close kinship, as individuals may frequently encounter distant or non-kin shortly after birth. Here, a recognition mechanism that allows individuals to assess relatedness based on similarity at a certain phenotype may be more dependable. These findings highlight that even within related taxa, kin recognition mechanisms are variable, and selection for discrimination is determined by the probability of encountering kin and non-kin, and the fitness outcomes of discriminatory behaviour in certain contexts or life-history stages.

In our study, relatedness did not affect the behaviour or physiology of pairs reared apart, nor were there any differences among relatives and non-relatives reared together. This suggests that recognition, at least in the context of mating decisions, may rely on familiarity alone. This differs from other social rodents, such as Belding ground squirrels *Spermophilus beldingi*, in which both early environment and genetic relatedness are important for kin recognition [22,50]. However, as different mechanisms may operate in different contexts, even within species, further investigation is now needed to determine whether familiarity is necessary for kin recognition in other contexts, such as cooperation or competition, and the extent to which kin discrimination occurs in such contexts.

In our study, inbreeding avoidance was examined with a factorial experiment that measured the responses of individuals to an assigned partner, which varied in familiarity and kinship. This represents the first opportunity to breed during sequential mate choice. Further studies, which permit females to sample multiple males of varying relatedness (or vice versa), might allow the strength of kin discrimination in mate choice to be assessed more closely, although such choice experiments may be difficult to execute. As females were not provided with an alternative male, we might have expected familiar pairs to ultimately breed. However, the continued abstinence observed among familiar pairs is consistent with that observed by subordinates within natural groups, which are often closely related [26,32].

As subjects were removed from the group and isolated before pairing, our findings reveal that recognition of familiar individuals is maintained outside the context of burrow system, ruling out mechanisms based on spatial cues or group membership *per se*. Instead, our results suggest that familiar individuals are recognized by some aspect of their phenotypes. In Damaraland and naked mole-rats, olfaction appears to be the primary modality for social communication [51,52], as in other rodents [53,54], and there is convincing evidence that several subterranean rodents discriminate conspecifics using olfactory cues (reviewed in [55]). However, other modalities should not be ruled out, and recognition may of course be multi-modal. In a broad range of taxa, kin discrimination has been documented based on chemical [22], acoustic [19], visual [48] or a combination of cues [56]. More studies are now needed to determine the sensory modalities of proximate recognition cues for incest avoidance. Whether Damaraland mole-rats can recognize individuals, or whether discrimination is based on a group signature that is shared among family members, also warrants further study.

Contrasts in the frequency of mating is reflected in female hormone profiles, showing that kin recognition operates at the physiological, as well as behavioural level. Apart from two exceptions, females paired with unfamiliar males showed high levels of sexual activity and increased their E2 and P4 levels after pairing, neither of which was observed in females paired with familiar males. The sustained elevation of E2 and P4 observed here is consistent with two mutually exclusive stages of reproduction that immediately follow ovulation, the luteal phase of the ovarian cycle and gestation [31] and therefore indicates recent ovulation. In mammals, ovulation can be induced by external stimuli related to mating, such as sensory cues from potential partners or coitus. It can also be spontaneous, occurring at regular stages of an oestrus cycle, independent of external stimuli [57]. Our finding that females’ first ovulation is contingent on preceding sexual activity shows that Damaraland mole-rats are induced ovulators. These results corroborate recent work from Voigt *et al.* [31] and is in contrast to the earlier suggestion that ovulation is spontaneous [58], though we cannot exclude the possibility that once reproductive activation is induced, ovulation may then occur spontaneously. In our case, the exception appears to prove the rule, as the female that ovulated after being paired with a familiar male also showed unexpectedly high levels of sexual activity. Likewise, during eight weeks of behavioural observations, no sexual activity was observed in the only female paired with an unfamiliar male that did not activate her reproductive axis during this time period. This is also supported by field evidence which shows that non-breeders become reproductive after dispersal [27,59]. In the wild, non-breeding female Damaraland mole-rats can remain solitary for extended periods after dispersal before encountering an unrelated male with whom to breed [59]. Yet, the chances of establishing a new breeding colony would be increased by readiness to breed [25]. In such circumstances, induced ovulation is adaptive, enabling reproduction soon after encountering a suitable partner [60].

Induced ovulation has important implications for the maintenance of reproductive skew in cooperatively breeding mammals that live in discrete family groups. In Damaraland mole-rats, anovulation of non-breeding females has been putatively attributed to social suppression by the breeding female [28,61,62]. However, if Damaraland mole-rats are induced ovulators, anovulation is the expected default state of the female reproductive axis until a suitable partner becomes available. In animals living in groups comprised of close relatives, this may only occur when females encounter a foreign male whose relatedness to them is likely to be sufficiently low. In such circumstances, it may thus be inappropriate to define non-breeding females lacking access to a mating partner as physiologically suppressed. This possibility is supported by several studies, including ours, which show that the E2 and P4 profiles of non-breeding females that lack a breeding opportunity remain low, even when the breeding female is absent [29,31]. Anovulation has also been observed in females that lack access to their usual breeding partner [31]. Studies that experimentally manipulate the family structure of groups, and the opportunities for females to breed with unrelated males, are needed to test whether, and how, breeding females suppress subordinate reproduction (for a rare example, see [33]).

All females paired with an unfamiliar male bred successfully, but there was no difference in offspring production between incestuous and unrelated pairs. Thus, there is no clear evidence in this study of postcopulatory inbreeding avoidance in Damaraland mole-rats, such as increased spontaneous abortion rates among related breeders. However, it is possible that foetal abortion is buffered in captivity, where food is provided ad libitum and exposure to parasites is limited. Advanced analyses that differentiate post-ovulation without fertilization from the early stages of pregnancy may provide more definitive conclusions regarding postcopulatory measures to reduce inbreeding. The fact that inbred foetuses are not aborted does not imply that inbreeding is not costly: across taxa, fitness costs are typically observed among inbred offspring rather than their parents [63,64]. Inbreeding costs must also be considered in balance with costs associated with inbreeding avoidance, such as missed mating opportunities [9] and inclusive fitness benefits of associating with kin [10]. Despite low rates of dispersal [25], thus limited opportunity to breed outside the group, Damaraland mole-rats overwhelmingly abstain from mating with group members [29], suggesting that inbreeding depression is sufficiently severe to select for strong inbreeding avoidance. Investigations of inbreeding depression, which compare fitness and fitness-associated traits among inbred and outbred individuals, make a compelling avenue for further study.

## 5. Conclusions

We have shown that in Damaraland mole-rats, kin recognition for incest avoidance operates through familiarity. Incest avoidance is maintained at the physiological level, with activation of the female reproductive axis requiring access to an unfamiliar male, but not necessarily an unrelated one. This study supports the growing body of work suggesting that early environment plays an important role in recognizing kin in a variety of species and behavioural contexts. Finally, we reveal important insights into how the ovulation is triggered, and the consequences of induced ovulation in social animals.

## Data Availability

Data relating to this article can be accessed in the Dryad Digital Repository [65]. Supplementary material is available online [66].
